# A retrospective study of different anterior and posterior approaches in the treatment of intervertebral discprotrusion at the cervicothoracic junction

**DOI:** 10.1097/MD.0000000000046050

**Published:** 2026-02-06

**Authors:** DeShuang Qi, TingFei Yan, FuLong Dong

**Affiliations:** a Department of Orthopedics, Linquan County People’s Hospital, Fuyang, Anhui, China; b Department of Orthopedics, The First Affiliated Hospital of Anhui Medical University, Hefei, Anhui, China; c Department of Orthopedics, Shanghai 411 Hospital, Shanghai, China.

**Keywords:** anterior decompression, cervicothoracic junction, intervertebral disc herniation

## Abstract

This study explores the efficacy of different anterior and posterior cervical spine surgeries in the treatment of cervical thoracic disc protrusion. A retrospective analysis was conducted on 31 patients with cervical thoracic intervertebral disc herniation who underwent surgical treatment at the Spinal Surgery Department of the First Affiliated Hospital of Anhui Medical University from January 2018 to May 2022. According to the surgical method, it is divided into anterior group and posterior group. Compare the general situation of 2 groups of patients, record the surgical situation and postoperative complications of the 2 groups. The Japanese Orthopedic Association (JOA) scoring system and visual analog scale (VAS) pain scale were used to evaluate the preoperative and postoperative functional recovery of patients. Patients in the posterior group had longer surgical time and more intraoperative blood loss compared to those in the anterior group. At 12 months after surgery, the VAS scores of both groups significantly decreased (*P* < .01), and the anterior group was significantly lower than the posterior group (*P* < .01). The JOA score is the opposite. The incidence of postoperative complications in the posterior group was significantly higher than that in the anterior group. If the segment of intervertebral disc herniation at the cervical thoracic junction is located above the sternum stem, anterior cervical surgery can avoid splitting the sternum, achieve good decompression and fixation, and be beneficial for restoring the physiological curvature of the spine at the cervical thoracic junction, achieving satisfactory clinical results.

## 1. Introduction

The cervicothoracic junction mainly refers to the spinal segment from C6 to T2 or the adjacent spinal segment.^[1]^ Cervical disc herniation is most common in the C3 to C7 intervertebral disc space. At present, there are few studies on patients with C7 to T1 disc herniation. With the development of medical imaging diagnosis, the reports of C7 to T1 disc herniation are increasing year by year, and the disc herniation at this site accounts for about 4% to 8% of all cervical disc herniation. Different from the general cervical disc herniation, the spinal canal at the cervicothoracic junction is relatively small, and patients often show obvious symptoms of spinal nerve root irritation or spinal cord compression,^[2,3]^ which often require surgical treatment. In the past, most of the literatures were about the treatment of vertebral fractures and tumors at this site, and there were few studies on disc herniation at this site.^[4,5]^ Because most of the compression objects come from the front, anterior surgery can directly relieve spinal cord compression and achieve satisfactory results.

Although spine surgeons are familiar with anterior cervical disc surgery for cervical disc herniation, it is difficult to expose the lesion through the anterior cervicothoracic approach because of the physiological curvature of the kyphosis, the complex adjacent anatomical structure and the obstruction of the manubrium sterni. For cervical intervertebral disc herniation at the cervicothoracic junction, the author analyzed the X-ray, CT, and MRI of the patients before operation, and selected the patients whose vertebral body at the level of sternal notch was located far from the diseased vertebral body for anterior cervical approach operation, and achieved good clinical results. The report is as follows.

## 2. Materials and methods

### 2.1. Clinical data

**Figure 1.**
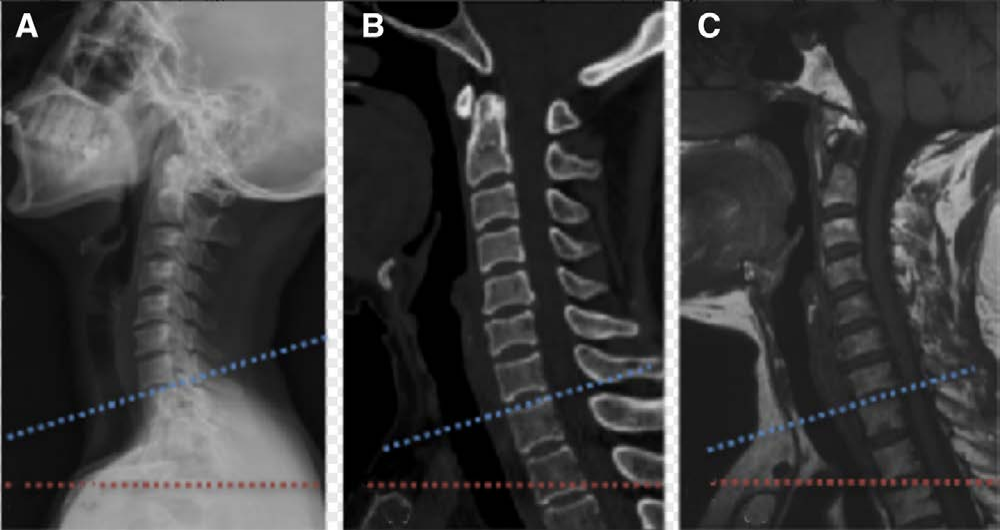
Cervical vertebra lateral radiography and MRI showed sternal notch is at the same level with C7/T1 intervertebral disc vertically.

From January 2018 to May 2022, we treated 31 patients with cervicothoracic junction lesions, including 29 patients with C7 to T1 disc herniation and 2 patients with T1 to T2 disc herniation. All patients had different degrees of neck and shoulder pain, upper limb pain and numbness, 6 cases were accompanied by muscle atrophy or muscle weakness of hand intrinsic muscles, and 9 cases were accompanied by cotton feeling in both lower limbs. Inclusion criteria: disc herniation at C7 to T2 level; and poor outcome after 3 months of conservative treatment. Exclusion criteria: patients with severe systemic disease who could not tolerate surgery; patients with tumor or infection; continuous ossification of the posterior longitudinal ligament of the cervical spine; fractures of the cervical vertebral body, pedicle, lamina, or spinous process caused by injury; and previous history of cervical surgery. Patients and their families gave informed consent to the study. Preoperatively, the procedure was evaluated by X-ray, CT, and MRI. All patients enrolled in the anterior group had a vertebral body subtended at the level of the sternal notch distal to the vertebral body below the herniated segment (Fig. 1). The patients were divided into anterior group (21 cases) and posterior group (10 cases) according to different surgical methods. This study was also approved by the institutional ethical review board of Linquan County People’s Hospital.

## 2.2. Surgical method

Anterior approach: the patient is placed in the supine position with a thin pillow over the scapula, so that the neck is in a neutral, slightly backward position. For patients with loose skin in the anterior cervical region, 2 to 3 elastic bandages were used to tighten the skin from the shoulders downward. All patients were exposed according to the routine anterior cervical surgery, and positioned under C-arm X-ray fluoroscopy. After exposing the diseased vertebral body and space, the cervical spreader was placed to open the diseased intervertebral space. Under direct vision, the diseased intervertebral disc was resected, the hyperplastic osteophyte at the posterior edge of the vertebral body was curetted, and the ossified posterior longitudinal ligament was curetted if there was ossification of the posterior longitudinal ligament, so as to completely relieve the compression of the anterior spinal cord. The appropriate internal fixation and fusion cage were selected according to the specific situation of the operation. The most distal level of fixation was T1. The specific surgical procedures were anterior cervical discectomy and decompression and fusion (ACDF) in 13 cases, anterior cervical corpectomy and decompression and fusion in 4 cases, skipping anterior cervical discectomy and decompression and fusion in 3 cases, and three-level anterior cervical hybrid decompression and fusion in 1 case.

Posterior approach: The patient is placed in the prone position and a longitudinal incision is made in the posterior midline of the neck. All patients were exposed according to the conventional posterior cervical surgery, and the total laminectomy was performed to decompress the spinal canal and expose the dural sac. After decompression, lateral mass screws were placed on both sides of the cervical vertebra. After the head was properly raised to restore the curvature of the cervical vertebra, prebent screws were placed on both sides for fixation. Of the 10 cases, 2 cases were fixed to the farthest segment T2. The specific surgical methods were single-segment posterior cervical spinal canal decompression and lateral mass screw fixation in 5 cases, multi-segment posterior cervical spinal canal decompression and lateral mass screw fixation in 4 cases, and skip posterior cervical spinal canal decompression and lateral mass screw fixation in 1 case.

## 2.3. Postoperative management

The patients were monitored for 8 to 12 hours after the operation, and the vital signs, the drainage volume of the incision drainage tube, and the sensory and motor changes of the limbs were closely observed. Antibiotics were routinely used for 24 hours after operation to prevent infection, aerosol inhalation was given to alleviate throat edema symptoms, and neurotrophic drugs were used to treat neurological symptoms. The drainage tube was pulled out 24 to 48 hours after operation when the drainage volume was <30 mL. If there are no special circumstances, they can sit or stand or get out of bed under the protection of neck brace on the second day after operation, and decide whether to cancel the protection of neck brace on the third month after operation.

## 2.4. Curative effect evaluation

The operation time, blood loss and complications were recorded. Postoperative outcomes were evaluated by the following methods: (Japanese Orthopaedic Association of Cervical Vertebrae (JOA of Cervical Vertebrae) score, Visual Analog Scale of pain (VASpain)^[6]^ recorded the functional recovery of patients before operation, the first week, 3 months, 6 months after operation, and the last follow-up. Within 1 week, 3 months, 6 months and more than 12 months after operation, the cervical spine was regularly reexamined by anteroposterior and lateral radiography, and if necessary, CT and MRI were performed to observe the fusion of cervical spine bone graft, whether there was fusion cage subsidence and pseudarthrosis formation.

## 2.5. Statistical analysis

SPSS 22.0 statistical analysis software was used for processing. Measurement data were expressed as mean ± standard deviation, and independent sample *t* test or repeated measures analysis of variance was used. Enumeration data were analyzed by Chi-square test. The difference was statistically significant when *P* < .05.

## 3. Result

### 3.1. Comparison of general conditions of the 2 groups

Gender, age, BMI, There was no significant difference between the 2 groups (*P* > .05) (see Table 1).

**Table 1:** Comparison of general conditions between the 2 groups.

Surgical method	Number of patients	Age	Gender	BMI	Follow-up time
Anterior approach	21	55.7 + 10.4	11/10	26.4 + 4.4	36.2 + 8.5
Posterior approach	10	63.4 + 12.0	7/3	23.9 + 3.1	37.9 + 7.9
*t*		−1.83	0.048	1.65	0.75
*P*		.078	.542	.113	.232

### 3.2. Comparison of intraoperative and postoperative data between the 2 groups

There were significant differences in operation time and intraoperative blood loss between the 2 groups (*P* < .05). Patients in the posterior group had longer operation time and more intraoperative blood loss (see Table 2).

**Table 2:** Comparison of intraoperative and postoperative data between the 2 groups.

Surgical method	Number of patients	Surgery duration (min)	Blood loss (mL)	Postoperative drainage (mL)	Length of stay
Anterior approach	21	110.7 + 28.4	50.0 + 23.9	38.8 + 23.4	8.4 + 1.5
Posterior approach	10	172.5 + 44.2	228.0 + 88.6	226.4 + 113.4	12.5 + 4.7
*t*		−4.71	−6.25	−5.18	3.106
*P*		.000	.000	.001	.002

### 3.3. Comparison of VAS and JOA scores before and after operation in the 2 groups

There was no significant difference in VAS and JOA scores between the 2 groups before operation (all *P* > .05). VAS scores were significantly lower in the 2 groups 12 months after operation (all *P* < .01), and the scores in the anterior group were significantly lower than those in the posterior group (*P* < .01). The opposite was true for JOA scores (see Table 3).

**Table 3:** Comparison of VAS and JOA scores before and after surgery between the 2 groups.

Surgical method	VAS score				JOA score			
	Preoperative	Postoperative	*t*	*P*	Preoperative	Postoperative	*t*	*P*
Anterior approach	6.6 ± 2.7	0.9 ± 0.5	8.624	.000	10.1 ± 2.6	15.7 ± 1.1	−9.939	.000
Posterior approach	6.4 ± 2.8	2.6 ± 1.7	7.067	.000	10.4 ± 2.1	14.2 ± 1.1	−6.326	.000
*T*	−0.321	−2.713			−0.632	2.346		
*P*	.832	.019			.479	.005		

JOA = Japanese Orthopedic Association, VAS = visual analogue scale.

## 4. Discussion

Cervical lesions are common in the upper and lower cervical vertebrae, and the cervicothoracic junction lesions are relatively rare, so there are few clinical studies, and the lesions in this area are easily misdiagnosed and neglected in clinic. The cervicothoracic junction is the transition region from the cervical spine to the thoracic spine, which generally includes the C6 to T2 spinal segments or their adjacent spinal segments. Because of the unique biomechanical characteristics of this segment, researchers are increasingly concerned about lesions located in this segment, especially C7 to T1 disc herniation. The herniated disc in this segment compresses the C8 nerve root, resulting in characteristic neurological signs and symptoms. The C8 nerve root is mainly composed of motor fibers and is responsible for innervating the intrinsic muscles of the hand. Patients often present with intrinsic muscle weakness of the hand,^[7]^ rarely paresthesia, and easily delayed diagnosis and treatment.^[8]^ The lower position of the shoulder may increase the mobility of the cervicothoracic junction and increase the incidence of disc herniation at the junction. Through this group of patients, we found that, compared with the general cervical disc herniation, cervical and thoracic junction disc herniation, although some patients have shown obvious symptoms of spinal nerve root irritation or compression,^[9]^ but the spinal cord compression on sagittal MRI may not be obvious, and at this time the herniated segment has obvious nerve root outlet stenosis and nerve root compression, which is easy to ignore in diagnosis. We speculate that there are 2 main factors leading to this change: one is that the posterior longitudinal ligament of this segment is narrow and thick, and it is difficult for the intervertebral disc to protrude in the middle, but it is easy to move laterally; the other is that there is no uncovertebral joint between C7 and T1, and the intervertebral disc can directly protrude into the C8 nerve root canal through the posterolateral side.^[10]^ Understanding the characteristics of disc herniation at the cervicothoracic junction will help in the diagnosis of disc herniation at this site. Therefore, clinicians should pay attention to this part of disc herniation, especially in patients with distant disc herniation and spinal stenosis at the same time, in order to avoid missed diagnosis and delayed treatment.

Anterior approach is still the first choice for the treatment of cervicothoracic disc herniation.^[11,12]^ The operation can directly relieve the compression of the cervicothoracic spinal cord or nerve root by the nucleus pulposus tissue protruding into the spinal canal after the rupture of the intervertebral disc, and obtain the most direct and thorough decompression effect. After the use of internal fixation, the operation segment can maintain the height and physiological curvature of the spine, obtain immediate stability after operation, and promote bone graft fusion.^[13]^ Because the cervicothoracic junction belongs to the stress concentration area of cervicothoracic flexion and translation,^[14,15]^ anterior fusion and fixation involving cervicothoracic junction does not have the same high risk of fusion failure^[16]^ as posterior fusion and fixation, so the risk of reoperation is low. However, due to the deep position of the intervertebral space, especially the low and downward inclination of the C7 to T1 intervertebral space,^[8]^ and the presence of sternum and thoracic structures (such as thoracic duct, pleura, thymus, brachiocephalic trunk and aortic arch), surgical exposure is very difficult and risky. How to evaluate the feasibility of anterior surgery and the possibility of passing through the sternum before implant placement is a problem faced by every spinal surgeon, and it is also an important key to the success of surgery. Obesity may be an important factor affecting the choice of surgical procedure. Toksoy et al^[17]^ reported that the median level of the cervical spine on standard lateral cervical radiographs was the sixth vertebra in patients with a high BMI (above 25) and the seventh vertebra in patients with a normal BMI (below 25). Lee et al believed that for obese patients with short neck, it may be necessary to split the sternum to expose the T1 vertebral body,^[18]^ but scholars at home and abroad found that the suprasternal notch of most patients corresponds to the T2/3 intervertebral space and below,^[13]^ so from the anatomical point of view, the application of anterior low cervical spine (without splitting the chest) can fully expose the C7 to T1 segment. A good surgical field was obtained, especially in patients with normal BMI.^[3]^ Because of the occlusion of the shoulder, the cervicothoracic junction is often not clearly displayed on the lateral X-ray film of the cervical spine. Except for some patients with degeneration, there are no specific signs on the X-ray film.

Through preoperative X-ray, CT and MRI imaging, we found that the horizontal line of the upper edge of the sternal manubrium in the anterior group of patients in this study was located in the lower 2/3 of the T2 vertebral body or the upper 1/3 of the T3 vertebral body, which was sufficient for exposing the T1 vertebral body and placing internal fixation.^[19]^ Therefore, all patients in the anterior group were treated with anterior surgery, and the operation was carried out smoothly, and there was no need to split the sternum during the operation. This study showed that the operation time and intraoperative blood loss in the posterior group were higher than those in the anterior group. After surgery, the JOA score of patients in the anterior group was higher than that in the nonsynchronous group, suggesting that anterior surgery can significantly improve the cervical spine function score of patients. At the last follow-up, the JOA scores of the patients in the same group were still higher than those in the non-same group, suggesting that the effect of anterior approach surgery on the recovery of neurological function was better than that of the posterior approach group, which may be due to the direct relief of spinal cord compression and the recovery of cervical curvature, thus improving the long-term effect and promoting the recovery of neurological injury. The authors suggest that for cervicothoracic disc herniation, careful preoperative evaluation of the relationship between the vertebral body and the herniated segment at the level of the sternal notch on X-ray, CT, and MRI is needed to select the appropriate surgical procedure. The anterior cervical approach, especially the non-split chest approach, can achieve good exposure of the herniated segment of the intervertebral space, thus achieving satisfactory decompression and immediate stable surgical results for those cases whose vertebral body corresponding to the level of the sternal notch is located far below the herniated segment on X-ray, CT, or MRI.

**Figure 2.**
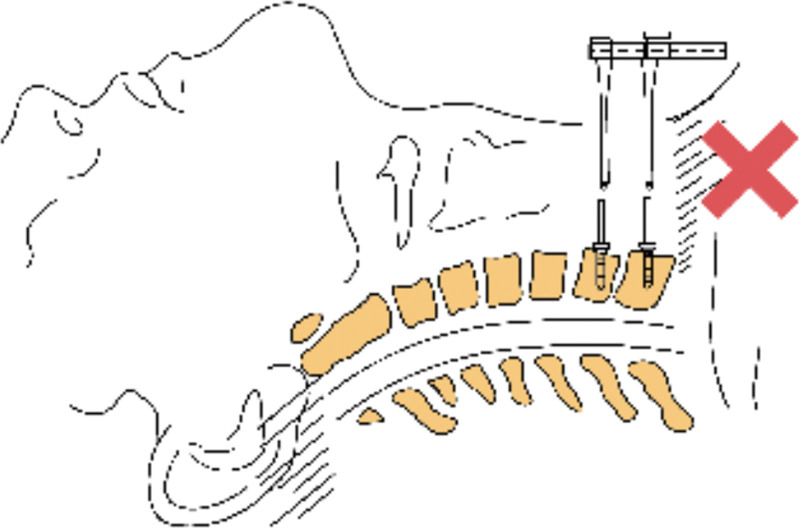
Due to the obstruction of the sternal stalk, it is often difficult to insert the spacer nail at the end of the conventional spreader, so a reverse spreader is used intraoperatively.

**Figure 3.**
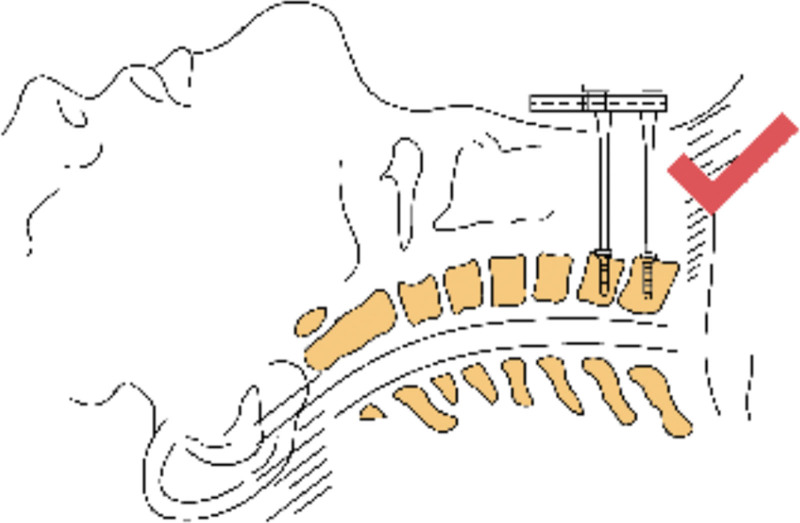
Due to the obstruction of the sternal stalk, it is often difficult to insert the spacer nail at the end of the conventional spreader, so a reverse spreader is used intraoperatively.

**Figure 4.**
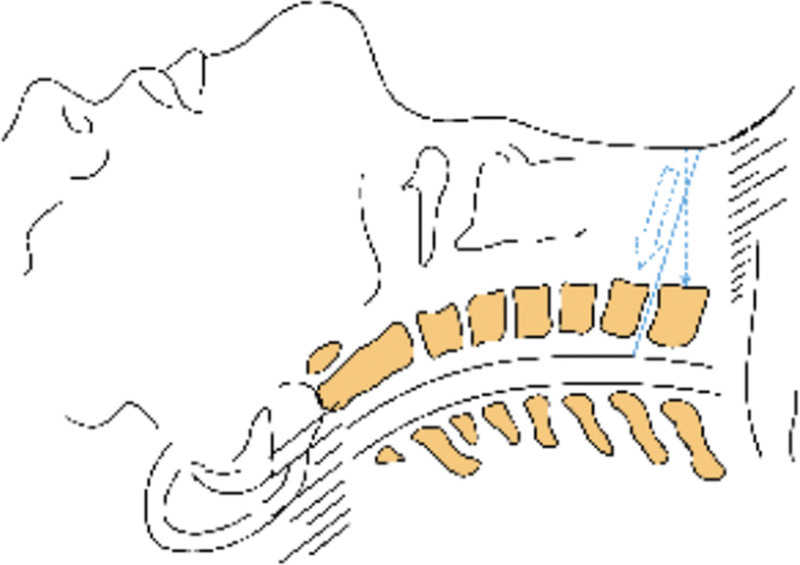
Imaginary line shows the horizontal direction of C7/T1 intervertebral disc, the arrow shows the direction of surgery, the double arrow shows the direction of skin incision.

**Figure 5.**
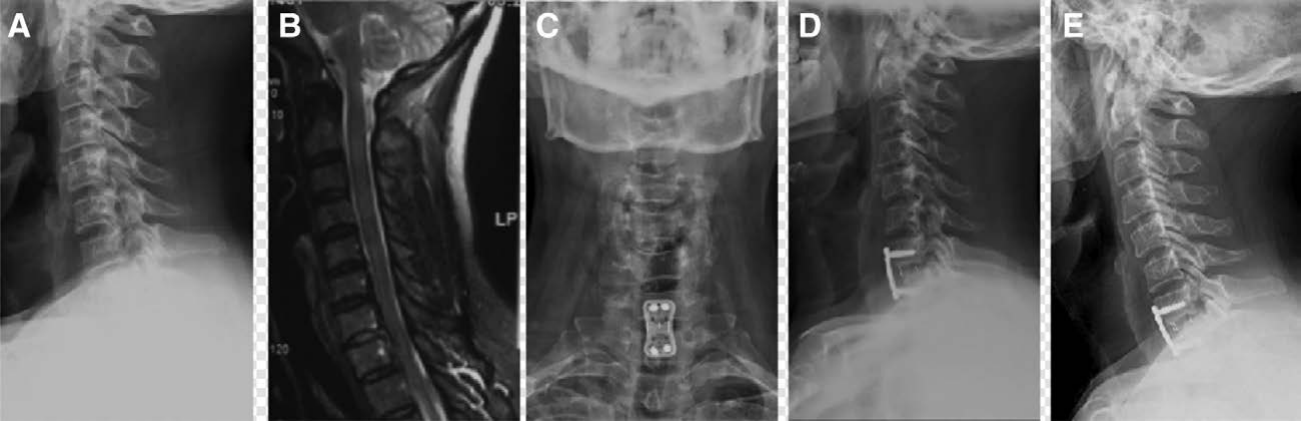
Case 1: (A) Lateral radiograph of cervical vertebra indicates that the physiological curvature of cervical vertebra becomes straight; (B) MRI sagittal plane of cervical vertebra shows I° spondylolisthesis of C7 vertebral body and protrusion of C7/T1 intervertebral disc; (C and D) the radiography showed that the internal fixation position was good after anterior decompression and fusion; (E) postoperative lateral radiography showed bone fused well at 12 months after operation.

**Figure 6.**
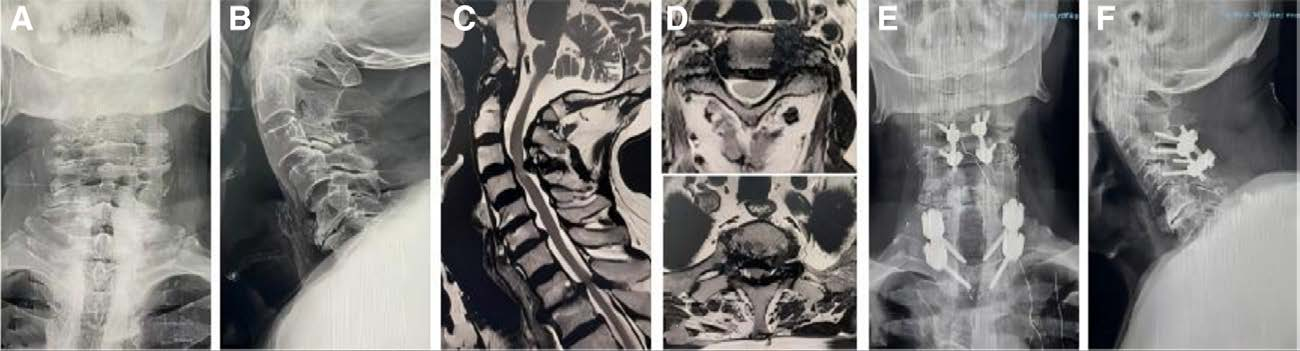
Case 2: (A and B) cervical X-ray shows cervical degeneration, osteophyte with intervertebral space stenosis; (C and D) MRI of cervical spine showed protruded intervertebral disc in C3/4, T1/T2 compression of the spinal cord; (E and F) postoperative X-ray images of jumping cervical spine posterior decompression and lateral block screw fixation suggest good internal fixation position.

The following points should be paid attention to in the treatment of cervical disc herniation at the cervicothoracic junction by anterior cervical surgery: Before the operation, the patient or his family should be instructed to use 2 to 4 fingers to repeatedly push the esophagus to the right side across the midline of the cervical vertebra at the space between the visceral sheath and the vascular sheath on the left side of the anterior neck, so as to make the esophagus and trachea in a relaxed state during the operation and reduce the physical consumption of the assistant during the operation. Is beneficial to the operation, improves the tolerance of the patient to the operation, and relieves the neck discomfort symptoms after the operation. In this study, 5 of 14 patients had hoarseness after operation, which was considered to be caused by excessive traction of recurrent laryngeal nerve during operation. Umana et al^[20]^ considered that the right recurrent laryngeal nerve had a high starting point and was obliquely medially and superiorly behind the carotid sheath, so the right recurrent laryngeal nerve was easily injured by the right low anterior approach to the lower cervical spine, while the left recurrent laryngeal nerve was close to the midline and located in the tracheoesophageal groove, requiring a short distance of traction during the operation. Therefore, the left low anterior approach is less likely to cause recurrent laryngeal nerve injury during the operation, so it is recommended to try the left approach to avoid recurrent laryngeal nerve injury during the operation for cervicothoracic junction lesions. We believe that most spine surgeons are more familiar with the right approach than left approach, and the right approach is more conducive to the operation, and the right oblique incision of the lower cervical spine can be extended downward during the operation. The recurrent laryngeal nerve can be clearly exposed through the right approach, and the right recurrent laryngeal nerve can be pulled downward and inward when exposing the lower cervical vertebra, and the right recurrent laryngeal nerve can be pulled to the SISU when exposing the upper thoracic vertebra, so as to reduce the tension of the right recurrent laryngeal nerve, avoid the injury of the recurrent laryngeal nerve, and avoid the accidental injury of the thoracic duct.^[21]^ Due to the occlusion of the shoulder, there may be misjudgment of the protrusion gap in the intraoperative fluoroscopic positioning, which may lead to the serious consequences of the wrong decompression segment. Therefore, care should be taken for multiple times of fluoroscopy during the operation to ensure the accuracy of positioning. Due to the obstruction of the sternal handle, it is often difficult to embed the distraction nail at the end of the conventional distractor, so the reverse distractor should be prepared before the operation (see Figs. 2 and 3). After anesthesia, the patient usually places a thin pillow on the scapula, so that the cervical vertebra is in a slightly backward position, which is conducive to obtaining a clear surgical field of vision. Considering the physiological radian of the kyphosis and the low and downward inclination of the C7 to T1 intervertebral space, the T1 vertebral body should be placed at an angle of 20° from the head to the tail as far as possible when placing the distraction nail in the lower cervical and thoracic vertebrae (see Fig. 4), to ensure that the needle is inserted into the vertical vertebral body, which is beneficial to the smooth embedding of the reverse distractor in the next step. One typical case of anterior and posterior approaches surgery was shown in Figures 5 and 6.

However, there are some limitations in this study. First of all, our county-level hospital belongs to primary medical institutions, and the limited number of cases may affect the reliability of the results; Secondly, the study only adopts single-center design and lacks longitudinal data support. In the future, we will continue to improve the construction of departments to expand our influence, so that patients can get medical services nearby, which can not only avoid the waste of treatment time, but also accumulate more clinical experience.

## 5. Conclusion

In view of the fact that there are many structures in the anterior part of the spine at the cervicothoracic junction, such as blood vessels, bones, nerves and joints, which make it difficult to expose the anterior cervicothoracic segment, our research group evaluated the preoperative imaging data of patients with disc herniation at the cervicothoracic junction, when the preoperative X-ray, CT, and MRI suggested that the corresponding vertebral body at the level of sternal notch was located. The extended anterior cervical incision without sternotomy can achieve good exposure, good decompression and immediate and stable surgical results.

## Acknowledgments

We thank all colleagues in the Department of Orthopedics, the Linquan County People’s Hospital, The Shanghai 411 Hospital, The First Affiliated Hospital of Anhui Medical University, for their help and support in this study.

## Author contributions

**Conceptualization:** DeShuang Qi.

**Data curation:** DeShuang Qi.

**Formal analysis:** DeShuang Qi, TingFei Yan.

**Funding acquisition:** FuLong Dong.

**Investigation:** FuLong Dong.

**Methodology:** FuLong Dong, TingFei Yan.

**Project administration:** FuLong Dong, TingFei Yan.

**Resources:** TingFei Yan.

**Software:** TingFei Yan.

**Supervision:** TingFei Yan.

**Validation:** FuLong Dong.

**Visualization:** FuLong Dong.

**Writing – original draft:** DeShuang Qi.

**Writing – review & editing:** DeShuang Qi.

## References

[1] Vickery JW Varas EE Abtahi AM. Crossing the cervicothoracic junction: a review of the current literature. Clin Spine Surg. 2022;35:451–7. 36447350 10.1097/BSD.0000000000001411

[2] Toll BJ Samdani AF Pahys JM Amanullah AA Hwang SW. Crossing the cervicothoracic junction in complex pediatric deformity using anterior cervical discectomy and fusion: a case series. Childs Nerv Syst. 2021;37:1957–64. 33730238 10.1007/s00381-021-05109-8

[3] Mostofi K Peyravi M Moghadam BG. Cervicothoracic junction disc herniation: our experience, technical remarks, and outcome. J Craniovertebr Junction Spine. 2020;11:22–5. 32549708 10.4103/jcvjs.JCVJS_102_19 PMC7274365

[4] Molina-Gil A Rodriguez LM Amorin-Diaz M. Tuberculous spondylodiscitis with a cervicothoracic spinal cord compression. Med Clin (Barc). 2021;157:e285–6. 32819768 10.1016/j.medcli.2020.07.021

[5] Balestrino A Gondar R Jannelli G Zona G Tessitore E. Surgical challenges in posterior cervicothoracic junction instrumentation. Neurosurg Rev. 2021;44:3447–58. 33754193 10.1007/s10143-021-01520-6

[6] Li H Ma Z Wang X. Comparative study of preoperative sagittal alignment between patients with multisegment cervical ossification of the posterior longitudinal ligament and cervical spondylotic myelopathy. Spine J. 2023;23:1667–73. 37355047 10.1016/j.spinee.2023.06.390

[7] Feigl GC Staribacher D Kuzmin D. Minimally invasive dorsal approach in the surgery of giant thoracic disk herniation: technical note and clinical case report. World Neurosurg. 2022;165:154–8. 35768057 10.1016/j.wneu.2022.06.097

[8] Post NH Cooper PR Frempong-Boadu AK Costa ME. Unique features of herniated discs at the cervicothoracic junction: clinical presentation, imaging, operative management, and outcome after anterior decompressive operation in 10 patients. Neurosurgery. 2006;58:497–501; discussion 497. 16528189 10.1227/01.NEU.0000197118.86658.A6

[9] Kim MS Gilbert ZD Bajouri Z. What does degeneration at the cervicothoracic junction tell us? A kinematic MRI study of 93 individuals. Eur Spine J. 2023;32:2425–30. 37148392 10.1007/s00586-023-07743-z

[10] Chakravarthy VB Hussain I Laufer I. Cervicothoracic junction instrumentation strategies following separation surgery for spinal metastases. J Neurosurg Spine. 2023;38:473–80. 36609370 10.3171/2022.12.SPINE22910

[11] Resnick DK. Anterior cervicothoracic junction corpectomy and plate fixation without sternotomy. Neurosurg Focus. 2002;12:E7. 10.3171/foc.2002.12.1.8 16212334

[12] Park EJ Jeong BG Min WK. Anatomical consideration for anterior approach of cervicothoracic junction: a computed tomography image analysis. Clin Orthop Surg. 2023;15:818–25. 37811505 10.4055/cios22394 PMC10551693

[13] Ryu DS Paik HK Ahn SS. Herniated discs at the cervicothoracic junction. World Neurosurg. 2018;118:e651–8. 30017762 10.1016/j.wneu.2018.07.017

[14] Politi LS Castellano A Papinutto N. Longitudinal quantitative magnetic resonance imaging in adrenomyeloneuropathy. Eur J Neurol. 2019;26:1341–4. 30932272 10.1111/ene.13959

[15] Chan AK Badiee RK Rivera J. Crossing the cervicothoracic junction during posterior cervical fusion for myelopathy is associated with superior radiographic parameters but similar clinical outcomes. Neurosurgery. 2020;87:1016–24. 32577734 10.1093/neuros/nyaa241

[16] Kong Q Li F Yan C. Biomechanical comparison of anterior cervical corpectomy decompression and fusion, anterior cervical discectomy and fusion, and anterior controllable antedisplacement and fusion in the surgical treatment of multilevel cervical spondylotic myelopathy: a finite element analysis. Orthop Surg. 2024;16:687–99. 38316415 10.1111/os.13994 PMC10925493

[17] Toksoy A Bektas F Eken C Ceken K Cete Y. Value of the swimming position and arm traction in visualizing the cervicothoracic junction over the standard lateral cervical X-ray. Int J Emerg Med. 2010;3:85–90. 20606816 10.1007/s12245-010-0159-y PMC2885263

[18] Lee JG Kim HS Ju CI Kim SW. Clinical features of herniated disc at cervicothoracic junction level treated by anterior approach. Korean J Spine. 2016;13:53–6. 27437013 10.14245/kjs.2016.13.2.53 PMC4949167

[19] Cho W Buchowski JM Park Y. P158. The surgical approach to the cervico-thoracic junction: can a standard smith-robinson approach be utilized? Clin Spine Surg. 2012;25:195S–6S. 10.1097/BSD.0b013e31821c2d60 21566532

[20] Umana GE Scalia G Ricciardi L. Surgical strategies and outcomes in degenerative myelopathy at the cervico-thoracic junction: a multicenter retrospective analysis. Eur Spine J. 2025;34:3453–63. 40676210 10.1007/s00586-025-09139-7

[21] Issa M Neumann JO Al-Maisary S. Anterior access to the cervicothoracic junction via partial sternotomy: a clinical series reporting on technical feasibility, postoperative morbidity, and early surgical outcome. J Clin Med. 2023;12:4107. 37373799 10.3390/jcm12124107 PMC10299693

